# Bi-Objective Optimization of Techno-Economic and Environmental Performance for Membrane-Based CO_2_ Capture via Single-Stage Membrane Separation

**DOI:** 10.3390/membranes15020057

**Published:** 2025-02-09

**Authors:** Nobuo Hara, Satoshi Taniguchi, Takehiro Yamaki, Thuy T. H. Nguyen, Sho Kataoka

**Affiliations:** Research Institute for Chemical Process Technology, National Institute of Advanced Industrial Science and Technology (AIST), Central 5, 1-1-1 Higashi, Tsukuba 305-8565, Ibaraki, Japan

**Keywords:** CCUS, CO_2_ capture, process design, life cycle assessment (LCA), multi-objective optimization, membrane separation, machine learning

## Abstract

Various factors need to be considered in process design optimization to implement the complex processes of CO_2_ capture, utilization, and storage (CCUS). Here, bi-objective optimization of single-stage CO_2_ membrane separation was performed for two evaluation indexes: cost and CO_2_ emissions. During optimization, the process flow configuration was fixed, the membrane performance was set under the condition of the Robeson upper bound, and the membrane area and operating conditions were set as variables. Bi-objective optimization was performed using an original algorithm that combines the adaptive design of experiments, machine learning, a genetic algorithm, and Bayesian optimization. Five case studies with different product CO_2_ purities in the constraint were analyzed. Pareto solutions were superior for case studies with lower product CO_2_ purities. The set of Pareto solutions revealed opposite directions for optimization: either (1) increase the membrane area to reduce CO_2_ emissions but increase costs or (2) increase power consumption and reduce costs but increase CO_2_ emissions. The implemented bi-objective optimization approach is promising for evaluating the membrane CO_2_ capture process and the individual processes of CCUS.

## 1. Introduction

Achieving carbon neutrality through CO_2_ capture, utilization, and storage is an urgent issue for building a sustainable society [1]. In the first stage of CCUS, CO_2_ is assumed to be separated and recovered from exhaust gas from power plants and factories that emit high concentrations of CO_2_. Conventionally, absorption, adsorption, membrane, and cryogenic separation are mainly studied for recovering carbon dioxide [2]. Although each method has distinct advantages and disadvantages, membrane separation is attracting attention as a promising CO_2_ capture method. Its main potential advantages are its continuous nature, ability to achieve separation without needing thermal energy, and miniaturizability [3,4,5,6,7,8].

The CCUS process equipment configuration and operating conditions should be designed and optimized to accelerate the practical application of CCUS processes, including CO_2_ recovery through membrane separation [9,10,11]. In process simulation, characteristic variables are used to calculate the material and thermal balance of each CCUS process, and an evaluation index is calculated within an evaluation boundary. Typical evaluation indexes include energy consumption, cost, and net present value. In addition to these conventional evaluation indexes, CO_2_ emissions are an essential evaluation index for CCUS, given that the original purpose of CCUS is to achieve carbon neutrality. They are as important as cost, which is an economic indicator. Therefore, bi-objective optimization must be performed using these two evaluation indexes. Pareto solutions, which are noninferior solutions that satisfy certain constraints, are generally obtained from multi-objective optimization, including bi-objective optimization. We reported the bi-objective optimization of cost and CO_2_ emissions for the absorption process in CO_2_ capture [12]. We subsequently studied the bi-objective optimization of cost and CO_2_ emissions for a continuous process with a larger evaluation boundary that connects the absorption process in recovery and methanation in utilization [13]. Bi-objective optimization clarifies the optimal process equipment configuration and operating conditions, thus potentially accelerating the implementation of the individual processes in CCUS.

Many reports have been published on process design and optimization for CO_2_ membrane separation [5]. Basic numerical calculation methods for membrane modules have been established using various models, such as co-current, counter-current, and cross-flow models, and some of them are currently available in commercial process simulators [14]. The first design issue is constructing a process flow that includes membrane separators, compressors, vacuum pumps, expanders, heat exchangers, and other components. The membrane module area, the permeance of each permeation component, and the operating conditions (for the compressor, vacuum pump, expander, and other parts) must be set to calculate the membrane separator. The characteristic dimensionless numbers for membrane process design are the pressure ratio in the membrane separator (Pr; ratio of feed-side pressure to permeate-side pressure, sometimes defined as a reciprocal), stage cut (ratio of permeate-side flow rate to feed-side flow rate), and overall process characteristics (including product CO_2_ purity and recovery). Regarding the number of stages in a membrane separator, the simplest setting has a single stage; however, multiple stages are required to achieve a CO_2_ concentration of 95% or higher and a recovery rate of 90% or higher, which are often set as standard product properties [15]. Therefore, multiple design elements should be determined in designing a CO_2_ membrane separation process.

Design optimization has been conducted via sensitivity analysis. In a fundamental study on the single-stage membrane process, Merkel et al. reported that the optimal CO_2_/N_2_ selectivity was between 20 and 40 and stated that the membrane area and cost increased with membrane selectivity [15]. They further analyzed multiple process flow configurations with fixed membrane performance and reported a trade-off between the membrane area and pressure ratio. In turn, this resulted in a trade-off between the membrane area and energy consumption for a two-stage counterflow/sweep membrane process. An optimal cost of USD 23/t-CO_2_ was reported, and the total recovery cost for energy consumption and membrane cost was minimized. Huang et al. examined the influence of the pressure ratio and membrane selectivity on membrane area and product CO_2_ purity [16]. They reported an optimal membrane selectivity of 20–40 (40–100) at a pressure ratio of 5 (10) of the feed side to the permeate side. They emphasized that membrane selectivity did not necessarily have to be excessively high to achieve the desired purity; the optimal selectivity of a membrane depended on the operation condition, especially the pressure ratio, so they should be balanced in design optimization. Ramasubramanian et al. reported the detailed modeling and cost sensitivity analysis of a two-stage membrane-based air-sweep process [17]. Sensitivity analyses were conducted on feed pressure and membrane-related parameters, such as selectivity, permeability, and price. Their results suggested that a recovery cost of USD 24/t-CO_2_ could be achieved at a membrane module price of USD 27/m^2^, feed-side pressure of 1 atm, membrane permeability of 3000 GPU, and membrane selectivity of 140. Li et al. performed a sensitivity analysis for membrane permeability and selectivity and stated that the membrane area and cost increased with increasing selectivity or decreasing permeability [18]. The authors also found a trade-off between energy consumption and membrane area from an analysis involving fixed membrane performance and varying pressure ratios. They further obtained a minimized cost of USD 28.7/t-CO_2_ from a sensitivity analysis on cost by changing the pressures for a two-stage membrane process. Xu et al. proposed a design method for single/two-stage membrane processes that resulted in 90% recovery and 95% purity, where membrane performance was set under the Robeson upper bound [19]. Sensitivity analysis was conducted on recovery cost by changing the membrane price, electricity price, and pressure, and a minimum cost of USD 25.2/t-CO_2_ was obtained. These sensitivity analyses showed a trade-off between membrane area and energy consumption, and design optimization was achieved for some of the design variables.

Systematic methods involving the simultaneous adjustment of multiple design variables have been reported for design optimization. First, optimization methods with fixed membrane performance have been proposed. In an early study, Sluijs et al. conducted a brute-force calculation in which the combination of the stage-cut and pressure ratio was changed to minimize the cost for single/two-stage membrane processes [20]. Micari et al. performed repeated calculations with different membrane areas and pressure settings to find the conditions leading to the minimum cost for a two-stage membrane process [21]. Second, optimization methods with variable membrane performance under the Robeson upper bound have been developed. Roussanaly et al. reported an attainable region approach to minimizing cost: purity was set for each stage in a multistage membrane process, and the process flow and design variables were selected [22]. Mat et al. reported an iterative calculation that started by setting the membrane selectivity and pressure conditions, followed by calculating the membrane area and the overall process to achieve 90% recovery and 95% purity [23]. Third, optimization methods that systematically explore the optimal process flow configurations using superstructures have been studied. A superstructure has been reported for optimizing process flow configurations, which considers all possible flow paths and systematically changes the selection of these flow paths and the distribution of flow rates at flow path branches. Superstructures are usually formulated using nonlinear programming or mixed-integer nonlinear programming, which can be solved by commercial solvers. Arias et al. optimized a multistage membrane process by changing the membrane area, power requirements, recycle stream location, and operating conditions to meet CO_2_ recovery and purity constraints at the minimum total cost [24]. They reported that the optimal process flow depended highly on the product CO_2_ purity. Chiwaye et al. optimized the cost of capture by considering using both CO_2_- and N_2_-selective membranes, reducing the membrane area by 46% and the capture cost by 14% relative to those obtained using CO_2_-selective membranes only [25]. Ramezani et al. optimized a process flow diagram and reported a minimum cost of EUR 42/t-CO_2_ for a polymeric membrane developed in their project [26]. Additionally, new optimization methods related to multi-objective optimization have been reported. Asadi et al. formulated an optimization problem for a two-stage process using a superstructure and performed multi-objective optimization for cost, required energy, and recovery rate using a multileader multi-objective particle swarm optimization algorithm [27]. Their identified lowest-cost process flow involved a counterflow two-stage membrane module with high permeability and a small membrane area, and a trade-off was observed between energy consumption and the CO_2_ recovery rate. Yuyama et al. extended evaluation boundaries to include the molecular structure of polymer membranes and studied optimization for two objectives (CO_2_ purity and recovery) considering membrane materials and processes simultaneously [28]. During optimization, a superstructure and an algorithm combining machine learning (ML) and Bayesian optimization were used to explore the optimal molecular structure that satisfied the specified product CO_2_ purity and recovery.

As mentioned above, various methods have been proposed for the design and optimization of process flow configurations, membrane areas, and operating conditions for CO_2_ membrane separation. However, the considered variables are mainly related to cost or energy consumption; no report has been published on optimization that explicitly uses CO_2_ emissions as an evaluation index or bi-objective optimization for cost and CO_2_ emissions. The design and operating conditions of membrane separation processes should be optimized more comprehensively using multiple variables to advance the implementation of membrane processes in CCUS.

In this study, focusing on a single-stage membrane process, we perform bi-objective optimization on CO_2_ membrane separation for cost and CO_2_ emissions. The process flow configuration was fixed, membrane performance was evaluated using permeability and the permeability ratio under the Robeson upper bound, and the membrane area and operating conditions were set as variables. The feed stream was postulated to be exhaust gas from a coal-fired power plant. The detailed process flow was evaluated, including dehydration from the feed exhaust gas, power recovery using an expander, and the heat exchangers. The evaluation indexes were (1) cost, which was calculated from the operational expenditure (OPEX) and capital expenditure (CAPEX), and (2) CO_2_ emissions; both were evaluated per unit weight of recovered CO_2_. Bi-objective optimization was performed using an ML-based multi-objective genetic algorithm Bayesian optimization (MLB-MOGABO), which is our original algorithm and combines the adaptive design of experiments (ADoE), ML, a genetic algorithm, and Bayesian optimization [13]. Five case studies with different product CO_2_ purities were analyzed. Bi-objective optimization analyses were performed for each case study, and the progress of MLB-MOGABO, the obtained Pareto solutions, and the impact of product purity on the Pareto solutions were thoroughly discussed.

## 2. Materials and Methods

The following sections present the elements of the bi-objective optimization process: boundary and process simulation settings, analyses of the evaluation indexes, the MLB-MOGABO algorithm, and the case study settings.

### 2.1. Boundary and Process Simulation Settings

**Figure 1:**
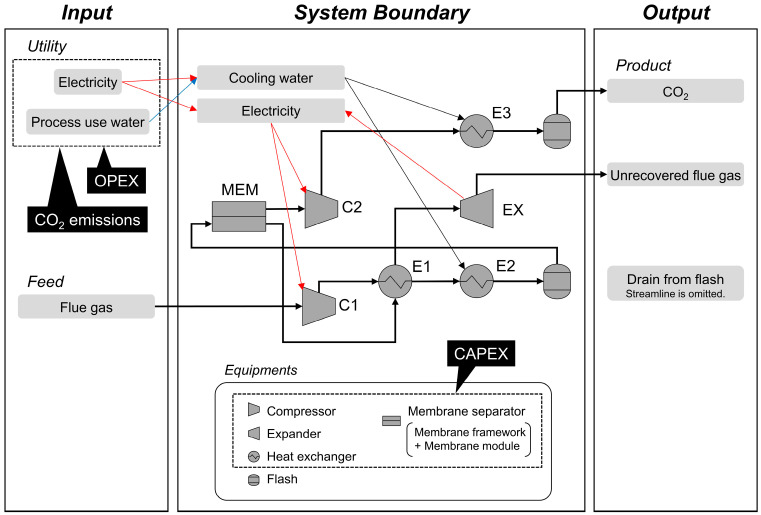
Process flow diagram and evaluation boundary.

The target process is a single-stage membrane process for recovering CO_2_ from flue gas, as shown in Figure 1. The feed flue gas, electricity, and process water were supplied to the system boundary, whereas product CO_2_, unrecovered flue gas, and drain from flashes were discharged. Table 1 presents the simulation equipment codes, types, and settings. The properties of the feed gas were set according to a previous study: flue gas from a coal-fired power plant with a 550 MWe net power output and a 79,200 kmol/h flow rate at 101 kPa and 57 °C and a composition consisting of N_2_ (68.8%), CO_2_ (13.5%), O_2_ (2.4%), and H_2_O (15.3%) [19]. The feed stream temperature into the membrane separator MEM was maintained at 57 °C by heat exchangers E1 and E2 after heating at the compressor C1. Power was recovered from the unrecovered flue gas by the expander EX. The heat transfer areas of the heat exchangers were calculated using the overall heat-exchange coefficient (U-value). The U-value was set at 1 kW/m^2^K for the liquid/liquid or steam/liquid fluid pair [29]. The U-value for the gas phase was determined by reference to the typical air-cooled heat exchangers: 0.005–0.1 and 0.1–0.3 kW/m^2^K for the 5–10 and 10–30 bar ranges [29]. For gas-phase cases with different pressures on each shell and tube side, or when one side contains liquid or steam, the U-values were calculated using the heat transfer resistance [13]. The whole process was designed and simulated using the process simulator PRO/II (version 2022, AVEVA) [30]. The margin of error in the calculation was set to 0.1% throughout the simulation.

The polymeric membrane separator MEM was calculated using the built-in calculation function in PRO/II based on the cross-flow method [14]. Compressors were installed on both the feed and permeate sides to optimize operation across a wide pressure range. From the MEM ideal separation factor (α*(CO_2_/N_2_)), membrane permeances for CO_2_, N_2_, O_2_, and H_2_O were calculated and selected as parameters, following previous studies. First, membrane permeances for CO_2_ and N_2_ were calculated to fulfill the relationship of the Robeson upper bound, assuming an effective membrane thickness of 100 nm [19,23]. For example, the CO_2_ permeance values were 1.28 × 10^−6^ and 1.74 × 10^−7^ mol/m^2^ s Pa (3840 and 519 GPU), with the α*(CO_2_/N_2_) at 50 and 100, respectively. Membrane permeance for O_2_ was then calculated by assuming that the ideal separation factor for CO_2_/O_2_ was 0.37 times that for CO_2_/N_2_ [19]. Membrane permeance for H_2_O was computed by assuming a 0.8 ideal separation factor for CO_2_/H_2_O [19].

### 2.2. Analyses of Evaluation Indexes

The selected evaluation indexes were the cost and CO_2_ emissions per unit weight of product CO_2_, and the evaluation parameters are shown in Table 2. For CAPEX estimation, the total module cost (C_TMi_) was first calculated using the following equation:

(1) $$\begin{array}{c} C_{TMi}=1.18\times \frac{{CEPCI}_{2021}}{{CEPCI}_{2001}}\times C_{p}^{0}{\left(2001\right)}_{i}\times F_{BMi}, \end{array}$$

where *CEPCI* is the chemical engineering plant cost index. The values for the base year (2001) and the annual value for 2021 were used, as shown in Table 2. *C_p_*^0^(2001)_i_ is the purchase cost of each piece of equipment for the base year (2001) and *F_BMi_* is the bare-module factor for each piece of equipment. Both were calculated using the settings shown in Table 1. For the evaluation of the membrane framework, the value for the base year (2014) was used as an exception. Then, the CAPEX per unit weight of product CO_2_ was calculated using the following equation:

(2) $$\begin{array}{c} CAPEX=\sum \left[\frac{{CRF\times C}_{TMi}}{Product\;flowrate\times Annual\;operation\;hour}\right], \end{array}$$

where *i* is an item in the list of equipment for CAPEX evaluation and *CRF* is the capital recovery factor, which was used to calculate the annual depreciation expenditure for each piece of equipment. *CRF* is determined assuming the service life of 25 and 5 years for general equipment and membrane modules, respectively, as shown in Table 2. For OPEX estimation, cooling water was obtained from utilities (electricity and process water), so the cost of cooling water was added separately to those utilities. Thus, the OPEX was evaluated using the following equation:

(3) $$\begin{array}{c} OPEX=\sum \left[Q_{j}\times {Price}_{j}\right], \end{array}$$

where j is an item in the list of utilities; *Q_j_* is the consumption amount per unit weight of product CO_2_ for each j, obtained from the process simulation and subsequent evaluations; and *Pricej* is the utility price of each j. Thus, the cost per unit weight of product CO_2_ was evaluated using the following equation:

(4) $$\begin{array}{c} Cost=CAPEX+OPEX. \end{array}$$

The other evaluation index was the CO_2_ emissions per unit weight of product CO_2_. Using the gate-to-gate life cycle assessment (LCA) method, we assessed all indirect sources of CO_2_ emissions from the utilities. Consequently, the total CO_2_ emissions were proportional to the utility consumption quantities, as described in the following equation:

(5) $$\begin{array}{c} {CO}_{2}\;emissions=\sum \left[Q_{k}\times C_{k}\right], \end{array}$$

where *k* is an item in the list of utilities; *Q_k_* is the consumption amount per unit weight of product CO_2_ for each *k*, obtained from the process simulation and subsequent evaluations; and *C_k_* is the CO_2_ emission factor per k.

### 2.3. MLB-MOGABO

In the MLB-MOGABO algorithm, the target process was evaluated and optimized in the following order: dataset generation, ML model building, exploration of the Pareto solution candidates, and verification. The above sequence was iterated based on the adaptive design of experiments (ADoE) by adding datasets and updating the models until satisfactory Pareto solutions were obtained [36,37]. All programs were written in Python 3.10.11, and the analyses were conducted on a personal computer equipped with a Core i7-1185G7 CPU (maximum frequency: 4.80 GHz).

The value ranges for generating the sample dataset were determined based on the figures in Table 3 for iteration 1. Sets of design variables were generated within the determined ranges using the D-optimal design for a specific number: 100 for iteration 1 and 10 for iteration 2 or later [38]. Process simulations were then performed using the set of generated design variables, followed by the analysis of the objective variables.

In ML model building, the main dataset was updated in every iteration by combining the sample and verification datasets in the previous iterations. The number of datasets was limited based on the ranges of the verification samples in the preceding iteration for iteration 51 or later to improve the coefficient of determination of the ML models. ML models were built in each iteration via ADoE using the main dataset under the settings in Table 4 using the scikit-learn library in Python [39]. For Y0, random forest classification (RFC) models were built by optimizing the hyperparameters using the out-of-bag method within the following ranges: n_estimators: 100–500, max_depth: 5–25, and max_features: 0.1–1 [40]. For Y1–Y4, Gaussian process regression (GPR) models were built using only the converged data by optimizing the Gaussian kernel through cross-validation [41].

In the exploration of the Pareto solution candidates, the elitist nondominated sorting genetic algorithm II was used, combined with the ML models built using the settings shown in Table 4 [42]. Regarding the minimization of the evaluation indexes Y3 and Y4, a lower confidence bound Y_Nac_ was used as the acquisition function for Bayesian optimization [43,44].

(6) $$\begin{array}{c} Y_{Nac}=Y_{Nave}-{SD}_{N}, \end{array}$$

where *Y_Nave_* and *SD_N_* are the predicted average and standard deviation values from the built ML models, respectively. As for the constraints, product CO_2_ recovery (Y2) was fixed at equal to or over 0.9, as widely used in previous studies. Exploration was performed using the Platypus-Opt library in Python, which has a population of 100 and a generation capacity of 20,000 [45]. From the above exploration, sets of the objective variables and corresponding design variables were obtained as Pareto solution candidates.

In the verification of the Pareto solution candidates, verification samples were selected under the maximum hypervolume. The hypervolume is an evaluation index for the extensity of Pareto solutions [45,46]. We analyzed the hypervolumes using the following reference point: (CO_2_ emissions, cost) = (2 t-CO_2_/t-CO_2_, USD 200/t-CO_2_). Five samples at most were selected for iteration 1 to iteration 50. Twenty samples were chosen for iteration 51 or later to improve the efficiency of exploring the Pareto solutions. Next, process simulations were performed by feeding the set of design variables from the selected samples, and the objective variables were obtained in the same manner as the generated sample dataset. The dataset of the verification sample was generated in this manner and combined with the main dataset. Pareto solutions were selected from the main dataset, and the number of Pareto solutions and the hypervolume were analyzed in every iteration. The ranges for generating the sample datasets in the subsequent iterations were determined from the ranges of the design variables of the previous verification samples: the minimum and maximum values of the design variables were multiplied by 0.8 and 1.2, respectively. The above iterations were implemented 70 times.

### 2.4. Case Study Settings

Bi-objective optimization was performed for five case studies to clarify the impact of product CO_2_ purity on the Pareto solutions. The product CO_2_ purity (Y1) in the constraint was changed from 0.9 to 0.5, as shown in Table 5. For all case studies, bi-objective optimization started with identical sample datasets in iteration 1.

## 3. Results

Bi-objective optimization of single-stage CO_2_ membrane separation was performed for two evaluation indexes: cost and CO_2_ emissions. The results were discussed under the following subsections: progress of bi-objective optimization; trends of Pareto solutions; relationship of product CO_2_ purity with α*(CO_2_/N_2_), P_h_, P_l_, and dimensionless numbers; relationship of product CO_2_ purity with membrane area and power consumption; and relationship of product CO_2_ purity with the area in the heat exchangers.

### 3.1. Progress of Bi-Objective Optimization

In this subsection, the progress of MLB-MOGABO-based bi-objective optimization for case study 1 is discussed as an example in the following order: dataset generation, model building, exploration of Pareto solution candidates, verification and selection of Pareto solutions, and iteration.

**Figure 2:**
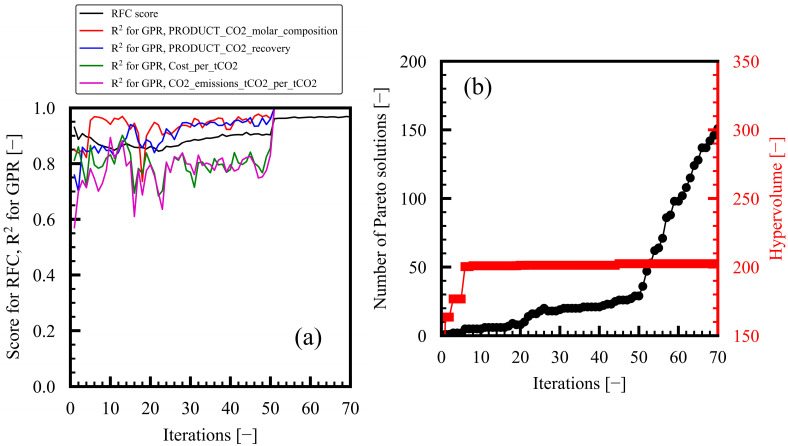
Progress of MLB-MOGABO-based bi-objective optimization for case study 1: (**a**) improvement in ML models and (**b**) changes in number of Pareto solutions and hypervolume.

Regarding dataset generation, 95 out of the 100 initial data points converged in iteration 1. In each iteration, more data points were added, and by the end of the 70th iteration, the total number of data points was 1434, out of which 1236 had converged. During RFC model construction for classifying the convergence Y0, the score initially decreased from 0.93 to 0.90 between iterations 1 and 50, as shown in Figure 2a. However, the score started increasing from iterations 51 to 70 due to the limiting of the amount of data based on the ranges of verification samples in the previous iteration. At the end of the 70th iteration, the score was 0.99. The most important features were in the following descending order of importance: C1 outlet pressure, MEM permeate-side pressure, MEM ideal separation factor, and MEM area. In the building of the GPR model to estimate objective variables Y1–Y4, the coefficient of determination (R^2^) improved to over 0.95 for Y1. The R^2^ values for the other objective variables were less than 0.9 at iteration 50, as shown in Figure 2a. By the end of the final (70th) iteration, the R^2^ values had exceeded 0.99 for all objective variables due to the limiting of the amount of data, as in the case of the RFC model. Consequently, the dataset expanded, and the prediction performance of the ML models improved as the iterations progressed.

**Figure 3:**
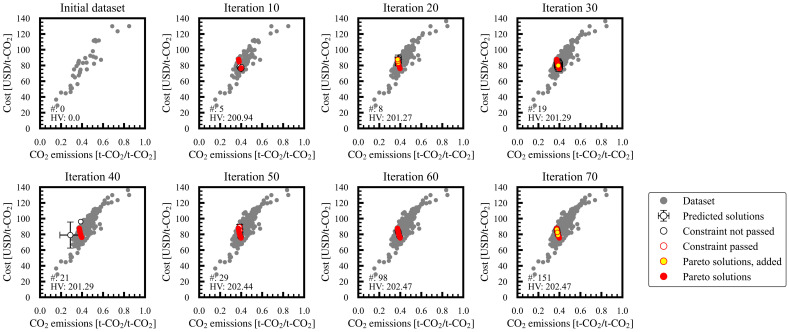
Regular observations of Pareto solutions for case study 1 in space of evaluation indexes. Number of Pareto solutions (#) and the hypervolume (HV) is shown in each figure.

Figure 3 shows the regular observations of the MLB-MOGABO iterations for case study 1. The unfilled black circles with error bars denote the GPR-predicted Y_Nave_ and SD_N_, the explored Pareto solution candidates. Subsequently, we performed process simulations using the design variables of the explored candidates, and the obtained data were plotted as follows: the unfilled red circles denoted the converged data that satisfied the constraints, the yellow-filled red circles were the newly obtained Pareto solutions in the iteration, the filled gray circles denoted the other dataset, and the filled red circles denoted all Pareto solutions in the preceding iterations. Two Pareto solutions were obtained in the initial dataset in iteration 1. As the iterations progressed, the Pareto solution candidates with higher probabilities of improving the evaluation indexes were explored based on Bayesian optimization. After verification, the explored candidates that satisfied the constraints and were not dominated by other solutions were adopted as Pareto solutions. In the next iteration, the sample data were added around the ranges of the design variables of the explored Pareto solution candidates in the preceding iteration based on Bayesian optimization.

**Figure 4:**
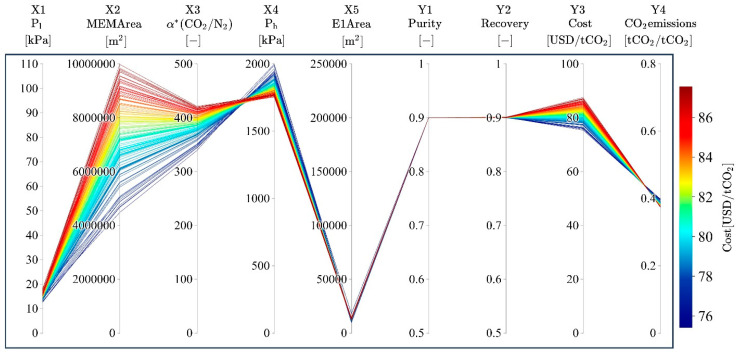
Visualized final Pareto solutions for case study 1.

Figure 2b shows the changes in the number of Pareto solutions and the hypervolume as the iterations progressed. The number of Pareto solutions became 5, 8, 19, 21, 29, 98, and 151 every 10 iterations. In general, the number of Pareto solutions increased as the iterations progressed, sometimes decreasing when newly obtained Pareto solutions dominated preceding ones. The hypervolume improved continuously with the progressing iterations, reaching 202.5 at the final (70th) iteration. The hypervolume almost reached a constant value after iteration 45. Consequently, the Pareto solutions were efficiently explored by MLB-MOGABO after iteration 70; the design variables and objective variables are visualized in Figure 4.

For case studies 2–5, bi-objective optimization started with identical sample datasets in iteration 1, as in case study 1. The optimization progressed similarly to that in case study 1; the progress of MLB-MOGAGO, the regular observations of the Pareto solutions, and the visualized Pareto solutions are shown in Appendix A. The number of Pareto solutions and the hypervolume for each case study obtained in the final (70th) iteration are shown in Table 5. The Pareto solutions were successfully analyzed via MLB-MOGABO-based bi-objective optimization for all case studies.

### 3.2. Trends of Pareto Solutions

**Figure 5:**
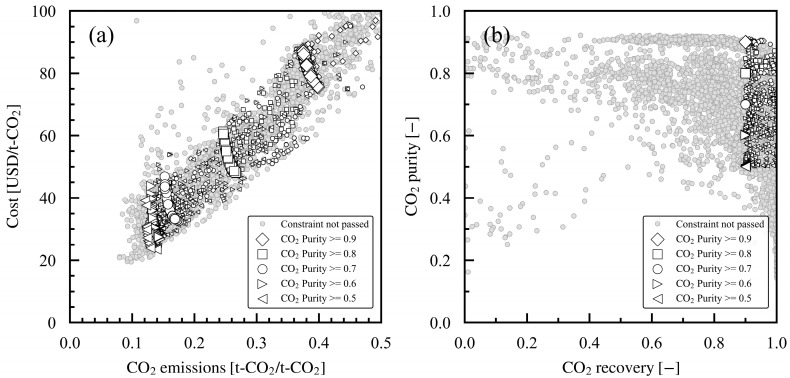
Pareto solutions for case studies 1–5 with different minimum product CO_2_ purities in constraint: (**a**) evaluation indexes (cost and CO_2_ emissions) and (**b**) constraints (CO_2_ purity and recovery). For each case study, the large symbols denote the Pareto solutions, and the small symbols denote the solutions that pass the constraints.

First, the overall trend of the Pareto solutions is discussed in this section for all case studies, as shown in Figure 5a. With a 0.9 product CO_2_ purity in the constraint, the CO_2_ emissions increased from 0.37 t-CO_2_/t-CO_2_ to 0.40 t-CO_2_/t-CO_2_ with a decrease in cost from USD 88/t-CO_2_ to USD 75/t-CO_2_, suggesting a trade-off. As the product CO_2_ purity in the constraint decreased from 0.8 to 0.6, the cost in the Pareto solutions decreased, and the minimum-to-maximum range changed to USD 48–61/t-CO_2_, USD 33–47/t-CO_2_, USD 27–44/t-CO_2_, and USD 23–39/t-CO_2_. The CO_2_ emissions also decreased overall, with the minimum-to-maximum range changing to 0.25–0.27, 0.15–0.18, 0.13–0.15, and 0.12–0.14 t-CO_2_/t-CO_2_. Overall, the cost and CO_2_ emissions in the Pareto solutions both decreased with the product CO_2_ purity in the constraint. Figure 5b shows the product CO_2_ purity and recovery rate as constraint variables. It shows the exploration of the Pareto solutions that satisfied a product CO_2_ recovery of 0.9 or higher and the product CO_2_ purity for each case study.

**Figure 6:**
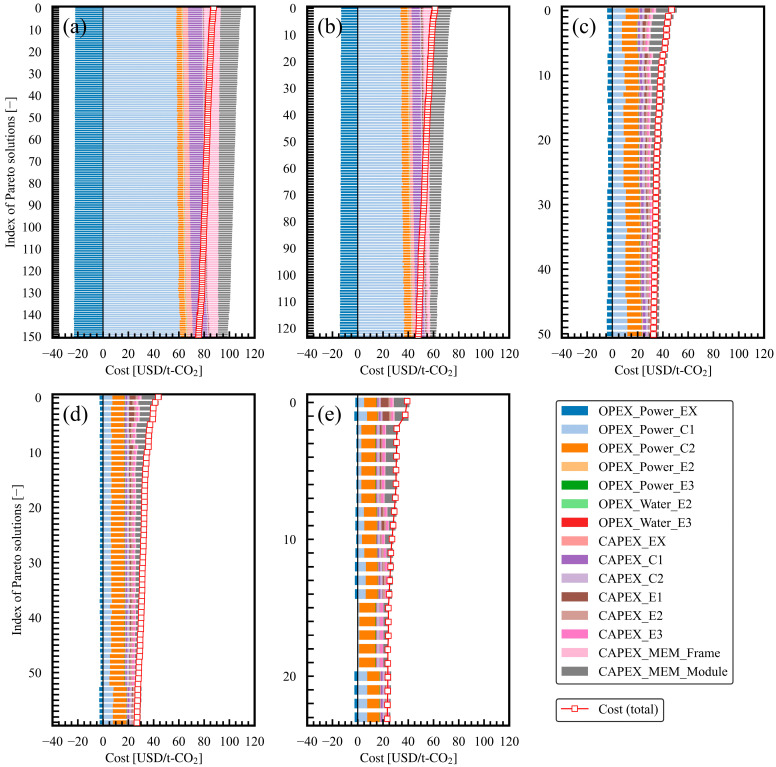
Details of cost in Pareto solutions for case studies 1–5 with different minimum product CO_2_ purities in constraint: (**a**) 0.9, (**b**) 0.8, (**c**) 0.7, (**d**) 0.6, and (**e**) 0.5 [−].

In evaluating the breakdown of indexes, nearly all CO_2_ emissions originated from electricity usage. CO_2_ emissions from process use water were much smaller than those from electricity usage. On the other hand, the costs included several factors. Details of the cost breakdown are analyzed and shown in Figure 6a–e for each product CO_2_ purity in the constraint. The sets of Pareto solutions with different product CO_2_ purities in the constraint were compared. The OPEX and CAPEX of C1 and EX, respectively, and the CAPEX of the membrane framework and membrane module increased with the increase in the product CO_2_ purity in the constraint. Furthermore, within each set of Pareto solutions with different product CO_2_ purities in the constraint, the higher-cost solutions had a small decrease in the compressor OPEX and higher proportions of membrane framework and module costs. The heat exchanger’s CAPEX accounted for a small proportion, but it became more noticeable in the Pareto solution sets with lower product CO_2_ purities in the constraint. The OPEX values of power and water for the heat exchangers were negligible. The relationship between the Pareto solutions and design variables is detailed further in the next section.

### 3.3. Relationship of Product CO_2_ Purity with α*(CO_2_/N_2_), P_h_, P_l_, and Dimensionless Numbers

**Figure 7:**
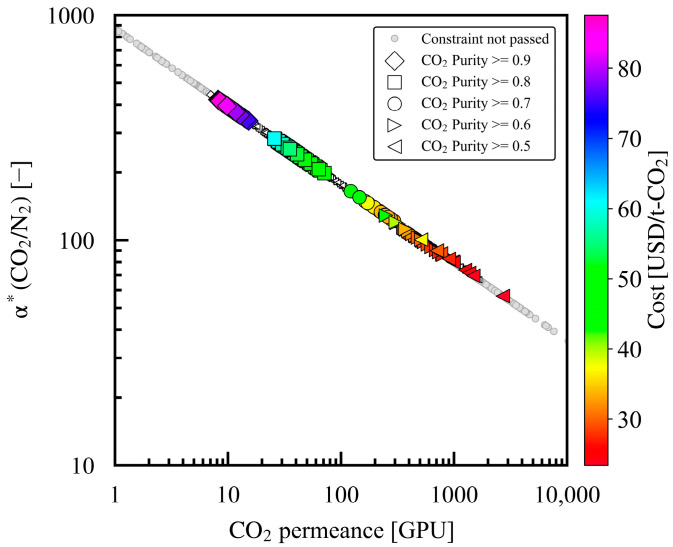
Optimized design variables α*(CO_2_/N_2_) and CO_2_ permeance for case studies 1–5 with different minimum product CO_2_ purities in constraint. The Pareto solutions are color mapped with the cost value of the evaluation index.

Figure 7 shows the design variable α*(CO_2_/N_2_) and the CO_2_ permeance in the entire dataset. The dataset plots were all along one line corresponding to the Robeson upper bound. A trade-off was evident between α*(CO_2_/N_2_) and CO_2_ permeance; as α*(CO_2_/N_2_) increased, CO_2_ permeance decreased. For case study 1, at a product CO_2_ purity of 0.9, the optimal values for α*(CO_2_/N_2_) ranged from 339 to 421. High α*(CO_2_/N_2_) values were required for case study 1, consistent with previous studies indicating the necessity of high-α*(CO_2_/N_2_) membranes for obtaining high product CO_2_ purities, especially in single-stage membrane processes [16,47]. The optimal ranges of α*(CO_2_/N_2_) decreased from 199–283 to 122–165, 86–129, and 56–101 as the product CO_2_ purity decreased from 0.8 to 0.5 for case studies 2–5.

**Figure 8:**
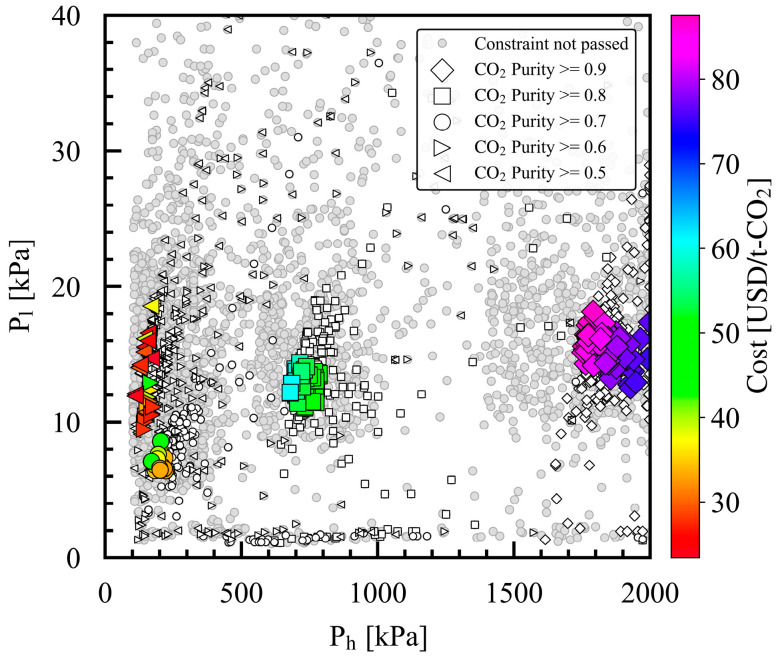
Optimized P_l_ and P_h_ design variables for case studies 1–5 with different minimum product CO_2_ purities in constraint. For each case study, the large symbols denote the Pareto solutions, and the small symbols denote the solutions that pass the constraints. The Pareto solutions are color mapped with the cost value of the evaluation index.

Figure 8 shows the P_h_ and P_l_ in the design variables. For case study 1, with a product CO_2_ purity of 0.9, the optimal values for P_h_ ranged from 1750 kPa to nearly 2000 kPa, which was the upper limit of the optimization range, as shown in Table 3. The optimal values for P_l_ ranged from 13 kPa to 18 kPa. As the product CO_2_ purity in the constraint decreased from 0.8 to 0.5, the optimal range for P_h_ decreased from 680–780 to 124–169. P_l_ decreased to below 10 kPa as the product CO_2_ purity in the constraint decreased from 0.9 to 0.7. P_l_ increased to nearly 20 kPa as the product CO_2_ purity further reduced to 0.6 and 0.5. Regarding the Pareto solution at the same product CO_2_ purity, the cost in the Pareto solutions increased as P_h_ decreased, but no strong relationship between P_l_ and cost was observed. The relationship between the Pareto solutions and operating conditions is discussed below.

**Figure 9:**
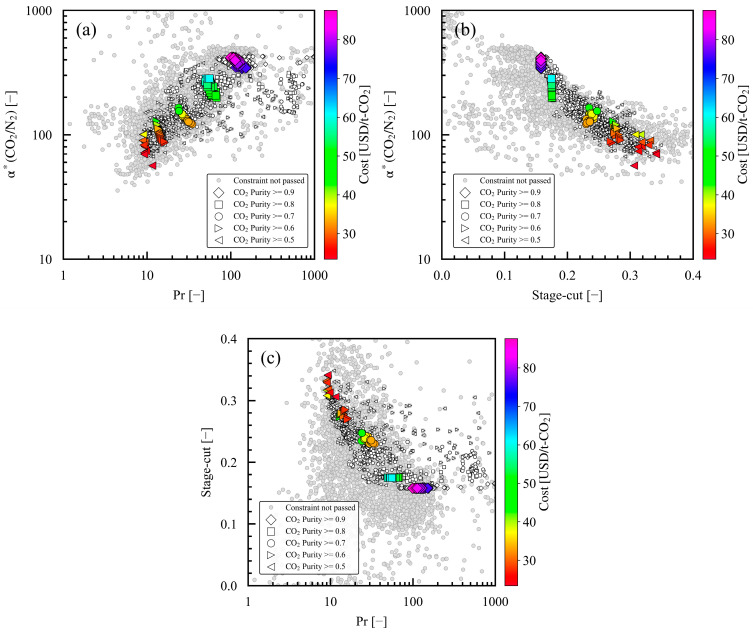
Dimensionless numbers for case studies 1–5 with different minimum product CO_2_ purities in constraint: (**a**) α*(CO_2_/N_2_) and Pr, (**b**) α*(CO_2_/N_2_) and stage cut, and (**c**) stage cut and Pr. For each case study, the large symbols denote the Pareto solutions, and the small symbols denote the solutions that pass the constraints. The Pareto solutions are color mapped with the cost value of the evaluation index.

Figure 9a shows the relationship between α*(CO_2_/N_2_) and Pr. Correlation with the Pareto solutions was higher for Pr, as shown in Figure 9a, than that for P_h_ and P_l_, as shown in Figure 8. Regarding the Pareto solutions at the same product CO_2_ purity, cost increased as α*(CO_2_/N_2_) increased and Pr decreased. A low Pr indicated low compressor power for pressure operation, consistent with the small decreases in the compressor OPEX in the higher-cost Pareto solutions, as discussed in Figure 6. As for the Pareto solutions at different product CO_2_ purities, both α*(CO_2_/N_2_) and Pr decreased with the decrease in the product CO_2_ purity in the constraint. This relationship aligns with a previous study showing that CO_2_ purity increases with Pr [16].

The relationship between α*(CO_2_/N_2_) and the stage-cut is shown in Figure 9b. Regarding the Pareto solutions at the same product CO_2_ purity, the effect of the stage-cut was not readily apparent. As for the Pareto solutions at different product CO_2_ purities, the stage-cut decreased with the product CO_2_ purity in the constraint. Pareto solutions may be explored to increase the stage-cut in case studies with lower CO_2_ purities in the constraint because the permeate-side CO_2_ fraction decreases as the stage-cut increases [48]. The relationship between the stage-cut and Pr is shown in Figure 9c, in which the dimensionless numbers both relate to the operating conditions. As the product CO_2_ purity in the constraint increased from 0.5 to 0.9, Pr increased from approximately 10 to about 100 and the stage-cut decreased from 0.3 to 0.16. In general, Pr should be increased and the stage-cut should be reduced to obtain a high-purity product CO_2_ concentration, and the trends of the Pareto solutions in this study are consistent with previously reported trends [16,49]. As described above, the Pareto solutions of the single-stage membrane process in this study had a high correlation with α*(CO_2_/N_2_), Pr, and the stage-cut.

### 3.4. Relationship of Product CO_2_ Purity with Membrane Area and Power Consumption

**Figure 10:**
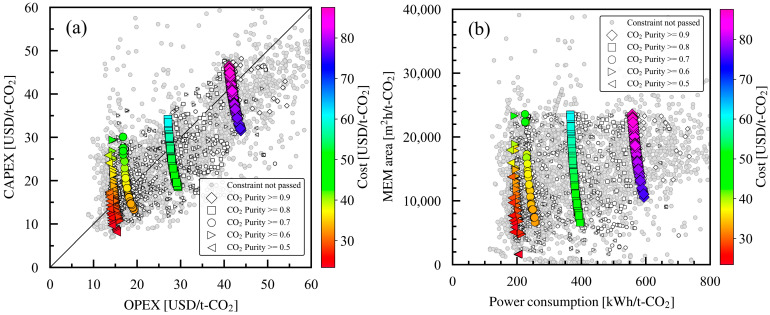
Comparison of components in Pareto solutions: (**a**) CAPEX and OPEX in Pareto solutions for case studies 1 to 5 with different minimum product CO_2_ purities in constraint and (**b**) membrane area and power consumption in Pareto solutions for case studies 1–5 with different minimum product CO_2_ purities in constraint. For each case study, the large symbols denote the Pareto solutions, and the small symbols denote the solutions that pass the constraints. The Pareto solutions are color mapped with the cost value of the evaluation index.

Cost as an evaluation index was further analyzed, especially two elements: the CAPEX derived from equipment and the OPEX derived from utilities. CAPEX decreased with an increase in OPEX (trade-off), as shown in Figure 10a. Additionally, the high-cost solutions were those with large CAPEX. Therefore, the orders of solutions in Figure 5a and Figure 10a are similar. Regarding the diagonal line between the CAPEX and OPEX axes in Figure 10a, CAPEX and OPEX are equal for the Pareto solution along this line; above the line are the Pareto solutions with large proportions of CAPEX, and below the line are the Pareto solutions with large proportions of OPEX. This study widely explored Pareto solutions on both sides of the diagonal line for CAPEX and OPEX.

The membrane area and power consumption were both analyzed and plotted on Figure 10b, whose values were the main elements of CAPEX and OPEX, respectively. For the same product CO_2_ purity in the constraint, a trade-off existed between membrane area and power consumption. The membrane area ranged from approximately 1600 m^2^h/t-CO_2_ to 24,000 m^2^h/t-CO_2_, little affected by the product CO_2_ purity in the constraint. On the contrary, power consumption decreased from 600 kWh/t-CO_2_ to 200 kWh/t-CO_2_ with a decrease in the product CO_2_ purity in the constraint from 0.9 to 0.5. The Pareto solutions with large membrane areas had large CAPEX values for both the membrane module and membrane framework, resulting in a large overall cost, as shown by the color map in Figure 10b. Furthermore, the Pareto solutions with high power consumption had large amounts of CO_2_ emissions in the final evaluation index. Therefore, a design policy with opposite directions was clarified in the bi-objective optimization for cost and CO_2_ emissions: (1) increase the membrane area to reduce CO_2_ emissions, although this will increase costs, or (2) increase power consumption to reduce costs, although this will increase CO_2_ emissions.

The relationships between the design variables can be understood in reference to previous studies. Xu et al. reported the relationship between membrane selectivity and membrane area for a single-stage membrane process with membrane performance under the Robeson upper bound [19]. They found that membrane area increased proportionally as selectivity increased at a fixed product CO_2_ purity. The current study also found that the membrane area increased in the cost-intensive Pareto solutions with an increase in α*(CO_2_/N_2_). Li et al. reported that an increase in membrane selectivity led to an increase in membrane area; however, increasing the feed pressure yielded the lowest cost point for a single-stage membrane process [18]. Moreover, similar to previous studies, the present work found that Pareto solutions could be explored by changing the balances between membrane area and pressure operation. Therefore, we extensively explored the Pareto solutions by adjusting the balance between membrane area and power consumption, which are dominant factors in the optimization of membrane process design.

### 3.5. Relationship of Product CO_2_ Purity with Area in Heat Exchangers

**Figure 11:**
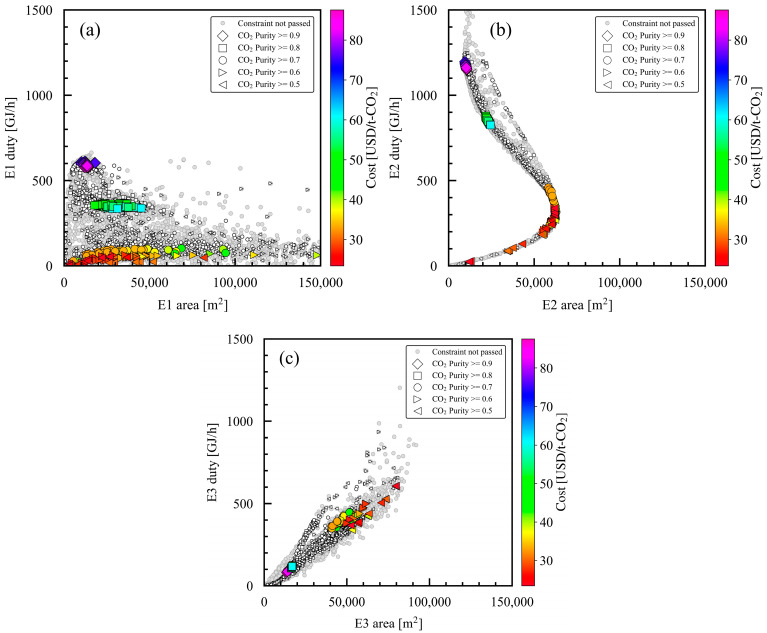
Optimized design variables for case studies 1–5 with different minimum product CO_2_ purities in constraint: (**a**) E1 duty and E1 area, (**b**) E2 duty and E2 area, and (**c**) E3 duty and E3 area. For each case study, the large symbols denote the Pareto solutions, and the small symbols denote the solutions that pass the constraints. The Pareto solutions are color mapped with the cost value of the evaluation index.

Although the heat exchangers accounted for a very small proportion of the overall cost and had negligible impact, they were optimized as follows. Figure 11 shows the heat transfer area and duty of E1, E2, and E3, including the design variable E1 area. Heat exchanger E1 increased power recovery in EX1 by increasing the temperature of the nonpermeate-side flow using the high-temperature flow after pressurization in the compressor C1. Regarding the E1 area, as shown in Figure 11a, the heat transfer area decreased, and the heat duty increased with the increase in the product CO_2_ purity in the constraint. P_h_ was higher for case studies with higher product CO_2_ purities in the constraint, as shown in Figure 8; therefore, the heat duty in E1 increased due to the high compression ratio in the compressor C1, and the heat transfer area was decreased by the high U-value in the process simulation settings. At the same product CO_2_ purity in the constraint, the E1 area could be optimized using the trade-off between the increased CAPEX caused by the large heat transfer area and the increased EX power recovery caused by the heating of the nonpermeate-side flow. Design variables were not set for E2 and E3, but they were optimized according to the simulation setting to maintain the temperature at 57 °C using cooling water. For heat exchanger E2, the heat duty increased, and the E2 area decreased with an increase in the product CO_2_ purity in the constraint for the same reason as explained for E1. For heat exchanger E3, the heat transfer area and heat duty increased proportionally with a decrease in the product CO_2_ purity in the constraint, which may have been caused by the increase in the total product flow rate. As stated above, we analyzed the Pareto solutions with different product CO_2_ purities in the constraint, discussed how they were affected by the design variables, and presented the breakdowns of CAPEX and OPEX.

## 4. Implications and Limitations

CO_2_ capture via membrane separation is potentially advantageous because it is continuous, does not require thermal energy for separation, and is compact; however, its design optimization has not been sufficiently investigated. In particular, bi-objective optimization of cost, which is important for process implementation, and CO_2_ emissions, which are essential for evaluating CO_2_ capture processes, is difficult due to the handling of the multiple design variables involved in the trade-off between the final evaluation indexes. This study is the first to report bi-objective optimization of cost and CO_2_ emissions for the membrane process. In this study, we analyzed Pareto solutions for the bi-objective optimization of cost and CO_2_ emissions by changing five design variables for the basic single-stage membrane process. Additionally, we conducted multiple case studies with varying product CO_2_ purities to elucidate the Pareto solutions and the influence of the design variables on the final evaluation indexes. Based on the knowledge obtained in this study, further optimal process design and new membrane development are expected to make membrane processes more competitive. Furthermore, MLB-MOGABO, a general algorithm for multi/bi-objective optimization, is expected to be applied to other CCUS and general chemical processes.

However, the target process was limited to a single-stage process in this study because the initial purpose was to examine bi-objective optimization for cost and CO_2_ emissions. Hence, some of the obtained knowledge may be inapplicable to the optimization of multistage membrane processes. Nonetheless, this study promotes the bi-objective optimization of multistage membrane processes, leading to progress in more practical design optimization of membrane processes in the future.

## 5. Conclusions

In this study, bi-objective optimization was performed on a single-stage membrane CO_2_ separation process for two evaluation indexes: cost and CO_2_ emissions. The original algorithm MLB-MOGABO was used for optimization, and dataset generation, ML model building, exploration of Pareto solution candidates, verification of Pareto solutions, and iteration based on ADoE were demonstrated. We conducted five case studies and analyzed the details of the Pareto solutions and the design variables at a fixed recovery rate and varying product CO_2_ purities. Bi-objective optimization for cost and CO_2_ emissions was performed in each case study, the impacts of target product CO_2_ purity on optimization were quantitatively analyzed, and the relationships of product CO_2_ purity with each design variable were clarified. According to our comparison of the case studies, cost and CO_2_ emissions both decreased with the product CO_2_ purity in the constraint. The OPEX and CAPEX derived from C1 and EX, respectively, decreased and the proportion of CAPEX from C2 increased with a decrease in the product CO_2_ purity in the constraint. The CAPEX of the membrane framework and module also decreased. Within the Pareto solutions for each case study, the α*(CO_2_/N_2_) and membrane area increased and Pr decreased with an increase in cost. However, these variables showed opposite changes with an increase in CO_2_ emissions. Higher-cost Pareto solutions showed larger membrane areas and higher CAPEX, and Pareto solutions with higher CO_2_ emissions showed higher power consumption and higher OPEX. In other words, process design optimization could be conducted in opposite directions: (1) increase the membrane area to reduce CO_2_ emissions, although costs will increase, or (2) increase power consumption and reduce costs, although CO_2_ emissions will increase. Further detailed analyses are expected for multistage membrane processes with various settings, such as membrane performance, process configuration, and electricity price as external factors.

## Figures and Tables

**Table 1:** Equipment codes, types, and settings for process simulation, and settings for CAPEX evaluation for process flow diagram in Figure 1.

Equipment Code	Equipment Type		Setting for Process Simulation	Setting for CAPEX Evaluation			
				Method	Capacity		
					Unit	Min.	Max.
C1, C2	Compressor		Adiabatic efficiency: 75%	CAPCOST [31], centrifugal, axial, and reciprocating	Fluid power, kW	450	3000
EX	Expander		Adiabatic efficiency: 75%	CAPCOST [31], radial gas/liquid expanders	Fluid power, kW	100	1500
E1, E3	Heat exchanger		Hot side: gas; cold side: gas; solved using minimum internal temperature approach (ΔT: 10 °C); U-value determined by pressure [13]	CAPCOST [31], floating head	Area, m^2^	10	1000
E2	Heat exchanger		Hot side: gas (product temperature: 40 °C); cold side: cooling water (inlet temperature: 30 °C, outlet temperature: 40 °C); U-value determined by pressure [13]	CAPCOST [31], floating head	Area, m^2^	10	1000
MEM	Membrane separator	Membrane framework	Calculated based on cross-flow model; pressure in both feed and permeate sides, membrane area, and permeance for all gas components used for calculation	Estimated based on previously published equation [22]; reference cost converted from EUR to USD at USD/EUR = 0.75	Area, m^2^	0	25,000
		Membrane module		Estimated by multiplying capacity and membrane module price	Area, m^2^	0	-

**Table 2:** Parameters for evaluating objective variables.

Parameter	Value	Unit	Remark
CAPEX			
Annual operation hour	8000	h/year	The annual production rate was calculated using the hourly production rate obtained from the process simulation multiplied by the annual operation hour
CEPCI_2001_	397.0	-	CEPCI for year 2001 [31]; used as base year
CEPCI_2014_	576.1	-	CEPCI for 2014 [32]; used as base year for evaluation of membrane framework
CEPCI_2021_	708.0	-	CEPCI for year 2021 [32].
CRF_st_	0.098	-	Capital recovery factor calculated from service life: 25 years; interest rate: 0.08; used as standard values
CRF_MemModule_	0.250	-	Capital recovery factor calculated from service life: 5 years; interest rate: 0.08; used for membrane module
Membrane module price	50	USD/m^2^	Changed in case studies
OPEX			
Electricity	0.0718	USD/kWh	Average price in US industrial sector in year 2021 [33]
Process use water	0.177	USD/1000 kg	[31]
CO_2_ emissions factor			
Electricity	0.656	kg-CO_2_/kWh	Calculated from the total CO_2_ emissions divided by the total electricity generation from coal, natural gas, and petroleum in the US in year 2021 [34]
Process use water	-	kg-CO_2_/1000 kg	Evaluated using SimaPro for water, completely softened [35]; value hidden as per terms and conditions of SimaPro

**Table 3:** Design variables and settings for bi-objective optimization.

Code	Design Variable	Unit	Setting for Bi-Objective Optimization			
			Range for Generating Initial Sample Dataset		Limit of Optimization Range	
			Min.	Max.	Min	Max
X1	MEM permeate-side pressure, P_l_	kPa	1	101	1	101
X2	MEM area	m^2^	100,000	10,000,000	1	10,000,000
X3	MEM ideal separation factor, α*(CO_2_/N_2_)	-	10	1000	10	1000
X4	C1 outlet pressure, P_h_	kPa	200	2000	101	2000
X5	E1 area	m^2^	10	1000	1	100,000

**Table 4:** Objective variables, settings for ML model building, and settings for bi-objective optimization.

Code	Objective Variable	Unit	Remark	Setting for ML Model Building		Setting for Bi-Objective Optimization	
				Dataset	Method	Objective	Constraint
Y0	Convergence	-	Convergence of process simulation (1/0)	All data were used	RFC	-	=1
Y1	Product CO_2_ purity	-	Molar concentration of CO_2_ in the product	Only the converged data were used	GPR	-	≥0.9, 0.8, 0.7, 0.6, 0.5
Y2	Product CO_2_ recovery	-	Recovery of CO_2_ in the product			-	≥0.9
Y3	Cost	USD/t-CO_2_	Cost per tCO_2_ in product			Minimize	-
Y4	CO_2_ emissions	t-CO_2_/t-CO_2_	CO_2_ emissions per tCO_2_ in product			Minimize	-
-	MEM ideal separation factor (CO_2_/N_2_)	-	Used as constraint in case studies 8, 9, and 10	-	-	-	≤50

**Table 5:** Settings and results of case studies.

Case Study Number	Setting	Result	
	Product CO_2_ Purity	Number of Pareto Solutions	Hypervolume
1	≥0.9	151	202.5
2	≥0.8	124	266.5
3	≥0.7	51	309.3
4	≥0.6	60	323.7
5	≥0.5	24	331.8

**Figure A1:**
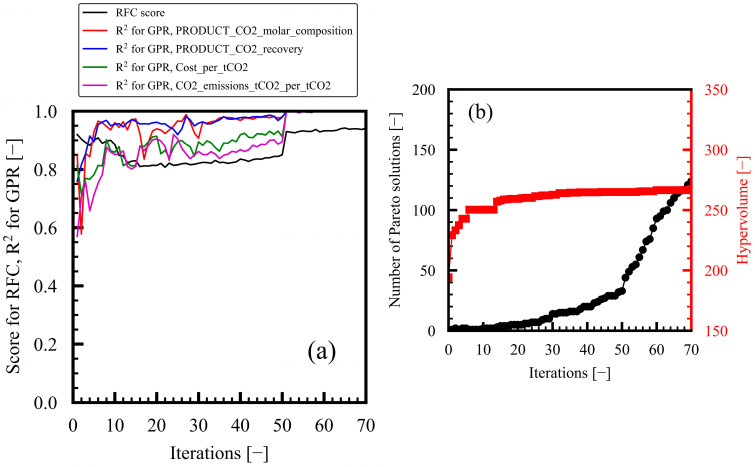
Progress of MLB-MOGABO-based bi-objective optimization for case study 2: (**a**) improvement of the ML models and (**b**) changes in the number of Pareto solutions and hypervolume.

**Figure A2:**
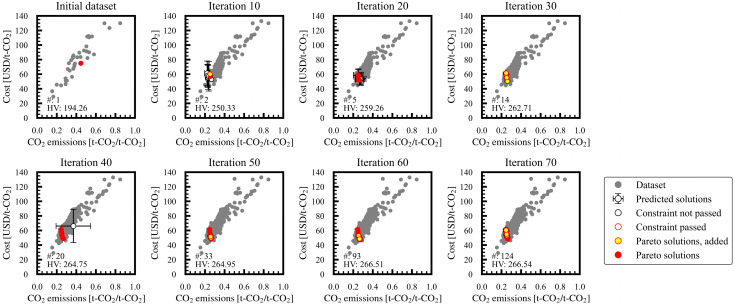
Regular observation of the Pareto solutions for case study 2 in the space of the evaluation indexes. Number of Pareto solutions (#) and the hypervolume (HV) is shown in each figure.

**Figure A3:**
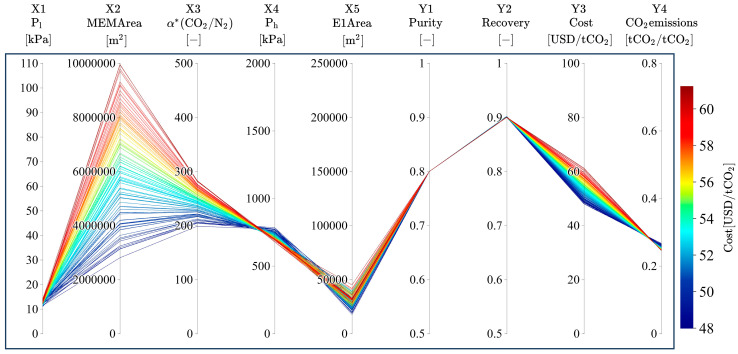
Visualized final Pareto solutions for case study 2.

**Figure A4:**
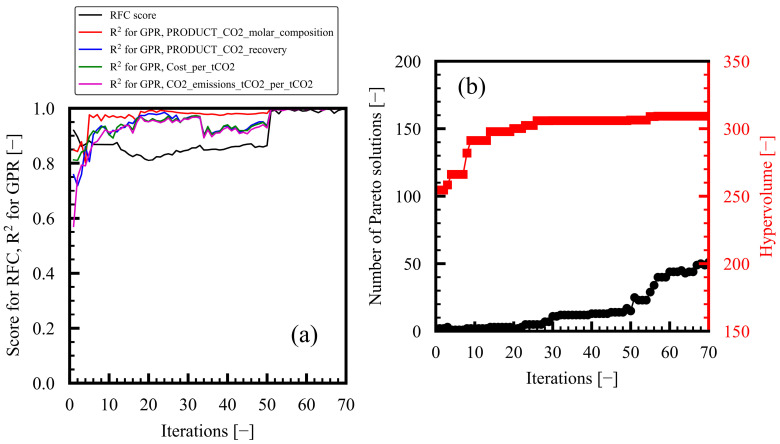
Progress of MLB-MOGABO-based bi-objective optimization for case study 3: (**a**) improvement of the ML models and (**b**) changes in the number of Pareto solutions and hypervolume.

**Figure A5:**
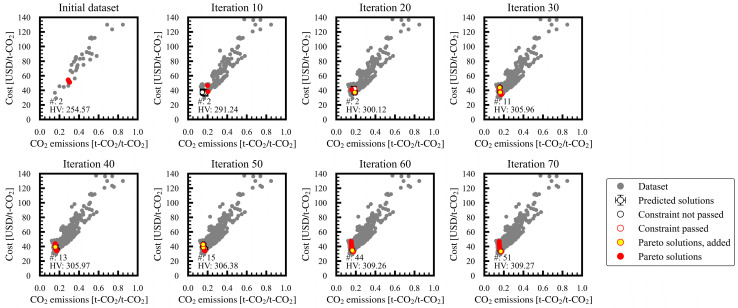
Regular observation of the Pareto solutions for case study 3 in the space of the evaluation indexes. Number of Pareto solutions (#) and the hypervolume (HV) is shown in each figure.

**Figure A6:**
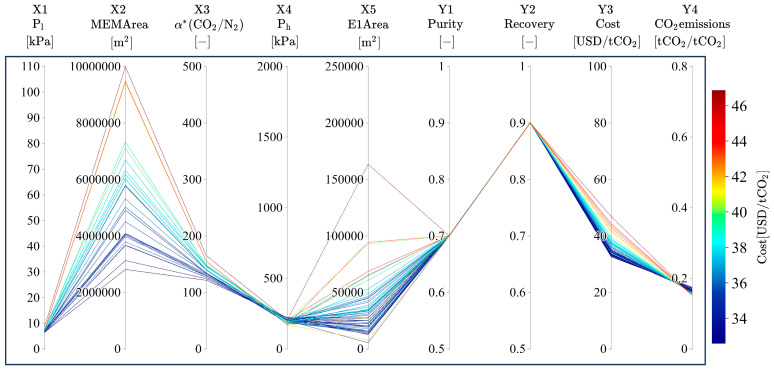
Visualized final Pareto solutions for case study 3.

**Figure A7:**
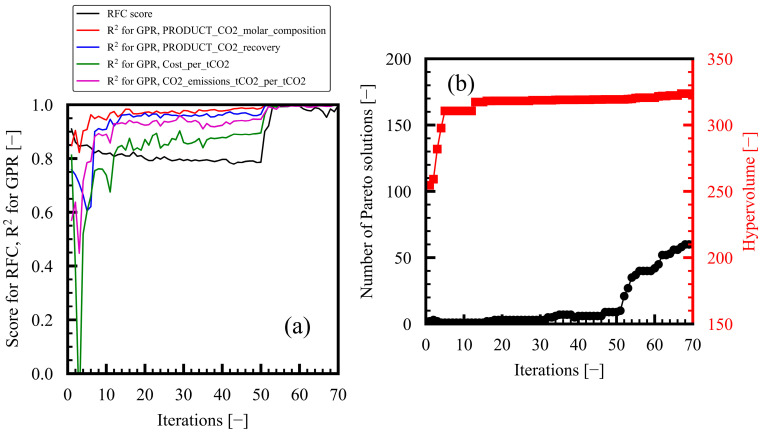
Progress of MLB-MOGABO-based bi-objective optimization for case study 4: (**a**) improvement of the ML models and (**b**) changes in the number of Pareto solutions and hypervolume.

**Figure A8:**
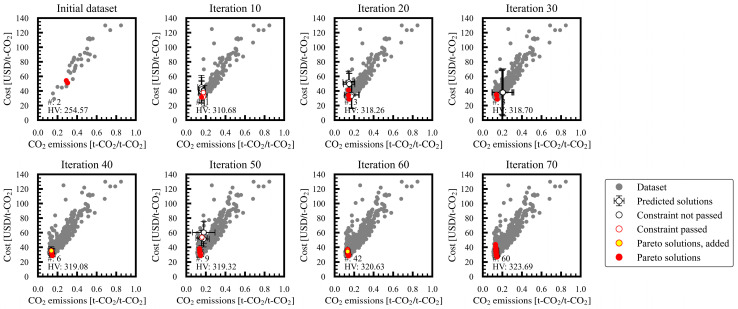
Regular observation of the Pareto solutions for case study 4 in the space of the evaluation indexes. Number of Pareto solutions (#) and the hypervolume (HV) is shown in each figure.

**Figure A9:**
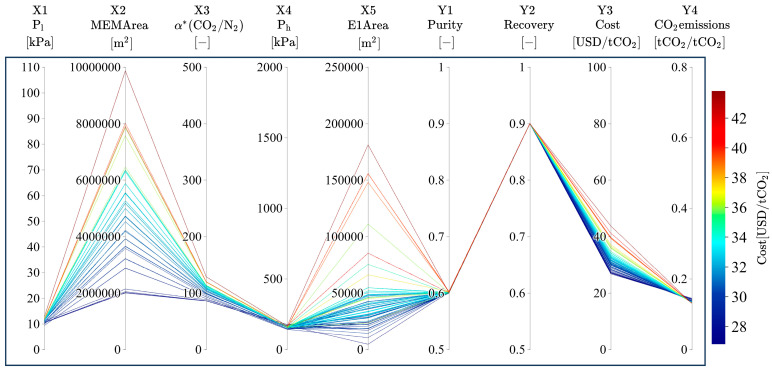
Visualized final Pareto solutions for case study 4.

**Figure A10:**
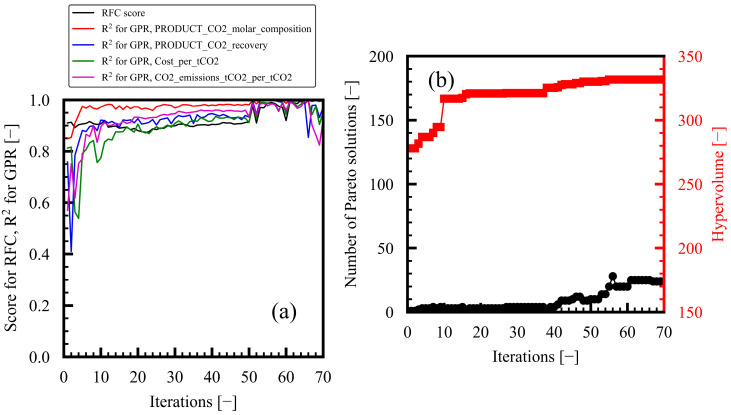
Progress of MLB-MOGABO-based bi-objective optimization for case study 5: (**a**) improvement of the ML models and (**b**) changes in the number of Pareto solutions and hypervolume.

**Figure A11:**
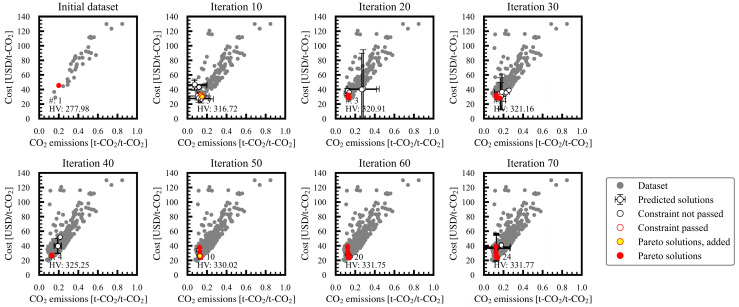
Regular observation of the Pareto solutions for case study 5 in the space of the evaluation indexes. Number of Pareto solutions (#) and the hypervolume (HV) is shown in each figure.

**Figure A12:**
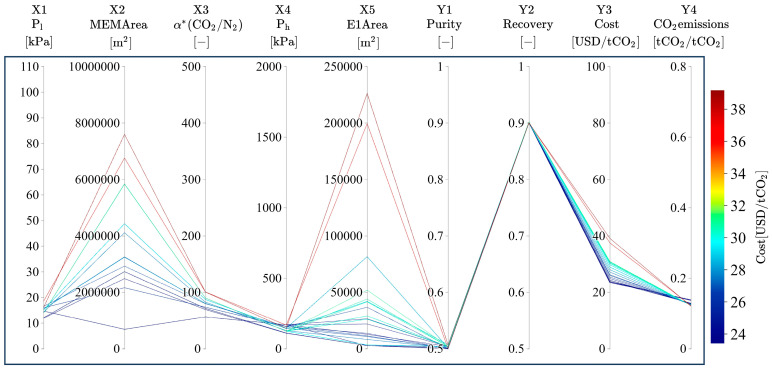
Visualized final Pareto solutions for case study 5.

## Data Availability

The data that have been used are confidential.

## Abbreviations

The following abbreviations are used in this manuscript:

Nomenclature	
CAPEX	Capital expenditure, USD/t-CO2
CEPCI	Chemical engineering plant cost index
CRF	Capital recovery factor
CTM	Total module cost, USD
FBM	Bare-module factor
OPEX	Operational expenditure, USD/t-CO2
Ph	Feed-side pressure, kPa
Pl	Permeate-side pressure, kPa
Pr	Pressure ratio (Ph/Pl)
R2	Coefficient of determination
U-value	Overall heat-transfer coefficient, kW/m2K
Equipment	
C	Compressor
E	Heat exchanger
Ex	Expander
MEM	Membrane separator
Acronyms	
ADoE	Adaptive design of experiments
CAPCOST	Capital equipment-costing program
CCUS	Carbon dioxide capture, utilization, and storage
GPR	Gaussian process regression
LCA	Life cycle assessment
ML	Machine learning
MLB-MOGABO	Machine learning-based multi-objective genetic algorithm Bayesian optimization
RFC	Random forest classification
